# T-SPOT.*TB *responses during treatment of pulmonary tuberculosis

**DOI:** 10.1186/1471-2334-9-23

**Published:** 2009-02-28

**Authors:** Samantha Ribeiro, Kelly Dooley, Judith Hackman, Carla Loredo, Anne Efron, Richard E Chaisson, Marcus B Conde, Neio Boechat, Susan E Dorman

**Affiliations:** 1 Instituto de Doenças do Torax/Hospital Clementino Fraga Filho/Universidade Federal do Rio de Janeiro, Rio de Janeiro, Brazil; 2 Center for Tuberculosis Research, Johns Hopkins University School of Medicine, Baltimore, Maryland, USA

## Abstract

**Background:**

Immune responses to *Mycobacterium tuberculosis *antigens could serve as surrogate markers of treatment response.

**Methods:**

Using the T-SPOT.*TB *assay and frozen peripheral blood mononuclear cells, we enumerated ESAT-6- and CFP-10-specific IFN-γ-producing T cells over time in pulmonary TB patients receiving directly observed treatment. T cell responses (measured as "spot forming cells" or "SFCs") were assessed prior to treatment and at 16 and 24 weeks of treatment.

**Results:**

58 patients were evaluated, of whom 57 were HIV seronegative. Mean (SD) ESAT-6, CFP-10, and summed RD1 specific SFCs declined from 42.7 (72.7), 41.2 (66.4), and 83.8 (105.7) at baseline to 23.3 (39.4, p = 0.01), 23.2 (29.4, p = 0.18), and 46.5 (59.5, p = 0.02) at completion of 24 weeks of treatment, respectively. Only 10% of individuals with a baseline reactive test reverted to negative at treatment week 24. For the group that was culture positive at completion of 8 weeks of treatment compared to the culture negative group, the incidence rate ratio (IRR) of ESAT-6, CFP-10, and summed RD1 specific SFC counts were, respectively, 2.23 (p = 0.048), 1.51 (p = 0.20), and 1.83 (p = 0.047). Patients with cavitary disease had mean ESAT-6 specific SFC counts that were higher than those without cavitary disease (IRR 2.08, p = 0.034).

**Conclusion:**

IFN-γ-producing RD1-specific T cells, as measured in the T-SPOT.*TB *assay, may be directly related to bacterial load in patients undergoing treatment for pulmonary TB. However, high inter-subject variability in quantitative results coupled with failure of reversion to negative of qualitative results in most subjects at treatment completion may limit the utility of this assay as a surrogate marker for treatment efficacy.

## Background

Progress in understanding the genome of *Mycobacterium tuberculosis *and human host responses has led to the rational development of diagnostic tests for tuberculosis (TB).[1] Early secretory antigenic target-6 (ESAT-6) and culture filtrate protein 10 (CFP-10) are secreted antigens encoded by region of difference-1 (RD1) of *M. tuberculosis*. These antigens elicit IFN-γ secretion by peptide-specific T cells.[1,2] Diagnostic tests have been developed to measure response T cell responses to these antigens.[3] T-SPOT.*TB *(Oxford Immunotec Ltd, Abingdon, Oxon, UK) is an ex vivo enzyme-linked immunospot (ELISPOT) assay that uses overlapping peptide panels to stimulate IFN-γ secretion by ESAT-6 and CFP-10-specific T cells.

Among individuals with active TB, the T-SPOT.*TB *test and similar ELISPOT tests have reported sensitivities of 83–97%. [1,4-10] In addition, compared with the tuberculin skin test, the T-SPOT.*TB *and similar ELISPOT tests have been shown to correlate more closely with extent of *M. tuberculosis *exposure in contacts of TB patients. [11-13]

However, the role of T-SPOT.*TB *in monitoring response to TB treatment is less well-established. A blood test that correlated well with severity of disease or treatment response might be useful as a surrogate marker in clinical trials of new TB treatments, to predict TB treatment failure and/or relapse, and to monitor treatment response in conditions such as extrapulmonary TB or childhood TB in which sputum is uninformative or not readily obtainable. In order to assess the potential utility of T-SPOT.*TB *for monitoring treatment efficacy, we evaluated the relationship between frequency of antigen-specific IFNγ-secreting T cells and treatment response in patients with pulmonary TB receiving directly observed therapy (DOT) in the context of a phase II TB treatment study. We also evaluated the relationship between SFC counts and markers of TB disease severity at baseline.

## Methods

### Setting and design

We performed a longitudinal cohort study of a diagnostic test, nested in a Phase II, single-center, double-blind TB treatment trial in Rio de Janeiro, Brazil [14]. The aims of the treatment trial were to evaluate and compare the antimicrobial activity of two daily TB treatment regimens administered during the first 8 weeks of treatment in patients with pulmonary TB. The control treatment arm was comprised of isoniazid, rifampin, pyrazinamide, and ethambutol (HRZE), and the experimental arm was comprised of isoniazid, rifampin, pyrazinamide, and moxifloxacin (HRZM). All drugs were given by directly observed therapy (DOT) five days per week. For the treatment trial, the primary endpoint was sputum culture status (positive or negative for *M. tuberculosis*) at completion of 8 weeks of treatment. All participants in the treatment trial were eligible for participation in the T-SPOT.*TB *diagnostic study. Blood for the T-SPOT.*TB *assay was collected just prior to initiation of TB therapy (week 0, baseline) and at 16 and 24 weeks of treatment. The treatment and diagnostic studies were approved by the ethics review boards of the Johns Hopkins University School of Medicine and the Hospital Universitario Clementino Fraga Filho in Rio de Janeiro, Brazil.

### Participants

Subjects were adults with smear positive, culture-confirmed pulmonary TB that was susceptible to all of the treatment drugs. Additional inclusion criteria were documented HIV status (positive or negative), no history of prior TB treatment, and Karnofsky performance status score 70 or greater. Subjects were excluded if they had only extrapulmonary TB, were being treated with HIV-1 protease inhibitors, were pregnant or breastfeeding, had AST > 3 times normal, total bilirubin > 2.5 times normal, creatinine > 2 times normal, hemoglobin < 7.0 g/dL, potassium < 3.5 mEq/L, or platelet count < 50,000/mm^3^. Upon initiation of the T-SPOT.*TB *substudy, all subjects enrolled into the parent treatment trial were co-enrolled in the TSPOT-TB study until completion of the treatment trial.

### Study evaluations

#### Enumeration of RD1-specific T cells

Whole venous blood was drawn into 8 ml CPT tubes (Becton Dickinson, Franklin Lakes, NJ) at weeks 0, 16, and 24 of TB treatment. Peripheral blood mononuclear cells (PBMC) were isolated by centrifugation, washed twice, resuspended in Recovery™ cell culture freezing media (Gibco, Invitrogen Corp., Carlsbad, CA), and frozen in liquid nitrogen until T-SPOT.*TB *testing. Just prior to testing, samples were thawed; twice, AIM V medium (Gibco, Invitrogen) was added and the samples were centrifuged. The PBMC pellet was resuspended in AIM V medium, and T-SPOT.*TB *assays using 8-well strips were performed according to the manufacturer's instructions.[15] For an individual study subject, all frozen samples were thawed and tested at one time, such that the final specimen (the 24 week specimen) was frozen for a minimum of 30 days. The assay included nil control, ESAT-6-derived peptide (Panel A), CFP-10-derived peptide (Panel B), and positive control wells. SFCs were enumerated on-site using a hand-held magnifying glass. As per the manufacturer's instructions, for ESAT-6 and for CFP-10, a test was scored as qualitatively reactive if either: (1) for nil control of 0–5 SFCs, the antigen well SFC count minus the nil control SFC count was ≥ 6; or (2) for nil control of > 5 SFCs, the antigen SFC count was ≥ 2 times the nil control SFC count. Overall, a T-SPOT.*TB *test was considered reactive if either ESAT-6 or CFP-10 was reactive, or considered nonreactive if both ESAT-6 and CFP-10 were nonreactive. The number of ESAT-6-specific SFCs was calculated by subtracting the nil control SFC count from the Panel A SFC count; the number of CFP-10-specific SFCs was calculated by subtracting the nil control SFC count from the Panel B SFC count. The resulting ESAT-6 and CFP-10 values were summated to give the total RD1 SFC count. Those wells with too-numerous-to-count SFCs were assigned a value of 400 which approximates the upper limit of quantification. The technologist performing the TSPOT-*TB *assays was not aware of patients' clinical or microbiological status.

#### Mycobacterial culture

For each subject, sputa were collected weekly through week 8, then monthly. Specimens were digested and decontaminated using the Nalc-NaOH method, and resuspended in 2.0 mL of phosphate buffer. Two 0.2 ml portions of resuspended sediment were plated onto Löwenstein-Jensen medium and incubated at 37°C for up to 8 weeks. For positive cultures, species identification was performed using conventional biochemical methods.

### Statistical analyses

To determine whether or not overall SFC counts changed over time with TB treatment, we compiled T-SPOT.*TB *results from baseline, 16 weeks of treatment, and 24 weeks of treatment. To compare SFC counts between those with positive and negative sputum cultures at treatment week 8 and to compare SFC counts between those with and without cavitary disease, we performed a longitudinal data analysis. To model the dependency resulting from repeated measures, and given that SFC counts represented overdispersed count data, the data were analyzed using a marginal model with negative binomial approach with generalized estimating equation methods (GEE).[16] We chose an independent correlation structure with robust standard errors for the GEE models, as quantitative statistical comparisons of fit showed that this correlation structure provided the best fit. Negative binomial regression models were used to analyze differences in SFC counts between groups at treatment week 24. Signrank tests were used to compare SFC counts before and after treatment. Two-sided alpha levels of 0.05 were used to determine statistical significance in all analyses. Statistical analyses were performed using STATA statistical software (StataCorp, College Station, Texas).

## Results

### Demographic and clinical characteristics of study participants

During the T-SPOT.*TB *study period, 73 individuals were enrolled in the TB treatment trial. Among these 73 subjects, 11 had no baseline T-SPOT.*TB *result and 4 had only a baseline T-SPOT.*TB *result; these 15 subjects were excluded from the analysis. The remaining 58 patients had at least two blood samples collected, including a baseline sample, and were included in the analysis.

Table 1 shows the baseline characteristics of the study population. Only one subject was HIV-infected (CD4 426 cells/mm^3^); ESAT-6 and CFP-10 spot forming counts for this patient were 14 and 99 at week 0, 4 and 77 at week 16, and 8 and 63 at week 24, resulting in a qualitatively reactive test at all time-points. Sensitivity of the T-SPOT.*TB *test, as measured by baseline reactivity of frozen samples, was 72.4% (95% CI 59–83%).

**Table 1:** Baseline characteristics of the study population.

	Total (n = 58)
**Characteristic**	
Mean age in years (SD)	33.4 (10.8)
Male (%)	36 (62.1)
Baseline weight in kg (SD)	55.7 (9.1)
Cavitary disease** (%)	41 (70.7)
Diabetes mellitus (%)	10 (17.2)
Assigned to HRZM treatment arm (%)	31 (54.4)
HIV-positive (%)	1 (1.7)†

*cavitary disease on baseline chest radiograph

† CD4 = 426 cells/mm^3^

SD, standard deviation

HRZE, combination treatment with isoniazid+rifampin+pyrazinamide+moxifloxacin.

### Association between SFC counts and severity of TB disease at baseline

To determine whether or not SFC counts were higher among those with more severe TB, we evaluated the association between SFC counts and two markers of disease severity at baseline – cavitary lung disease and low body mass index (BMI). For ESAT-6, those with cavitary disease had, on average, 2.08 times higher SFC counts than those without cavitary disease (95% confidence interval for the incidence rate ratio [IRR] 1.06 to 4.10, p = 0.034), but CFP-10 SFC counts did not differ between the two groups (p = 0.64). There was no association between baseline BMI and either ESAT-6 or CFP-10 SFC counts (p = 0.48 and 0.48, respectively).

### SFC counts over time during TB treatment

**Figure 1:**
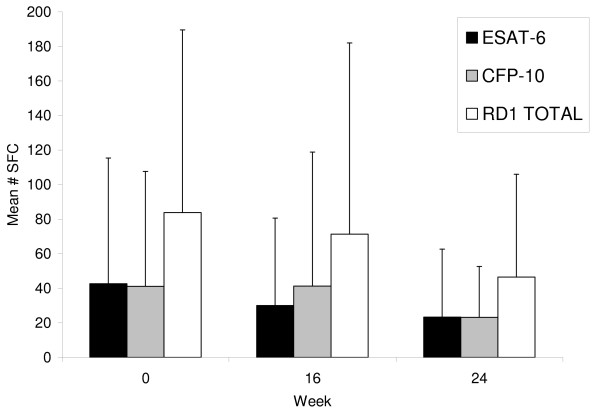
**Mean ESAT-6, CFP-10, and summed RD1 specific spot forming cells at weeks 0 (baseline), 16, and 24 for all study participants (n = 58)**. SFC, spot forming cells. Vertical bars represent standard deviations of the means.

As shown in Figure 1, mean SFC counts declined over 24 weeks for ESAT-6, CFP-10, and summed RD1. Mean (SD) ESAT-6 SFC declined by 22.2 (62.9) over 24 weeks (p = 0.01 by signrank test), but CFP-10 SFC declined, on average, by only 8.9 (41.2) over 24 weeks (p = 0.18).

Table 2 shows the percent of study subjects having a reactive qualitative result for ESAT-6, CFP-10, and RD1 at the three time-points. Using qualitative results, there were no significant trends over time, and no differences between percentages of subjects positive by ESAT-6 versus by CFP-10 at any of the time-points. Of 42 individuals who had a reactive baseline test, 40 were retested at week 24; of these 40, 4 (10%) had a non-reactive T-SPOT.*TB *test at week 24.

**Table 2:** Qualitative T-SPOT. *TB *results: % of subjects having a reactive test, stratified by stimulatory antigen and by culture results at completion of 8 weeks of TB treatment

		Week 0	Week 16	Week 24
**ESAT-6**				
	All subjects	56.9	58.9	53.7
	Culture positive	60.0	77.8	60.0
	Culture negative	56.3	55.3	52.3

**CFP-10**				
	All subjects	55.2	58.9	59.3
	Culture positive	60.0	77.8	60.0
	Culture negative	54.2	55.3	59.1

**RD1**				
	All subjects	72.4	78.6	75.9
	Culture positive	70.0	88.9	70.0
	Culture negative	72.9	76.6	77.3

### SFC counts among patients who were sputum culture positive versus negative at completion of 8 weeks of TB treatment

**Figure 2:**
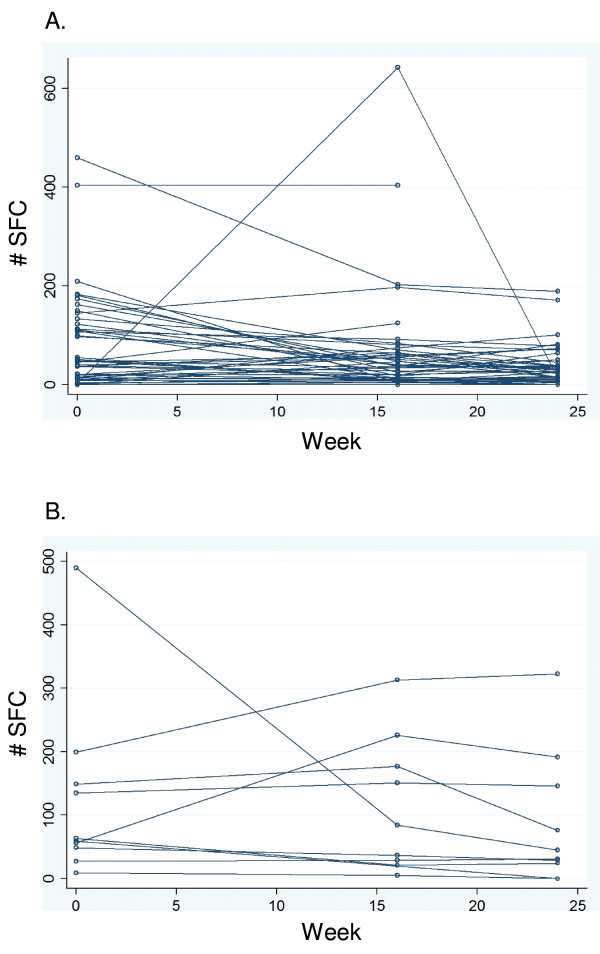
**Individual patient RD1 spot forming cell response profiles over 24 weeks of TB treatment for individuals having a negative sputum culture for *M. tuberculosis *at completion of 8 weeks of TB treatment (PANEL A, n = 48), and for individuals having a positive sputum culture at completion of 8 weeks of TB treatment (PANEL B, n = 10)**. SFC, spot forming cells.

**Figure 3:**
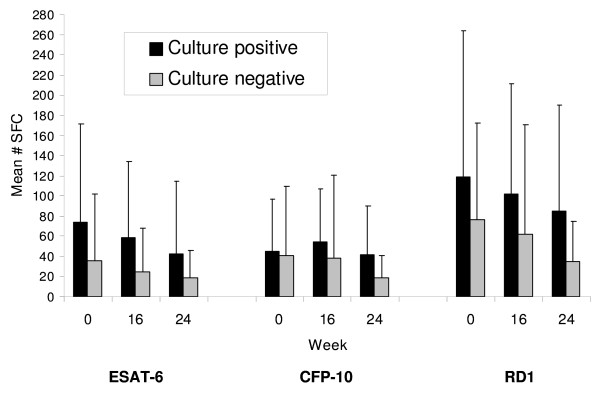
**Mean ESAT-6, CFP-10, and summed RD1 specific spot forming cells at weeks 0 (baseline), 16, and 24 for individuals having a positive sputum culture for *M. tuberculosis *at completion of 8 weeks of TB treatment (SOLID BARS, n = 10), and for individuals having a negative sputum culture at completion of 8 weeks of TB treatment (HATCHED BARS, n = 48)**. SFC, spot forming cells. Vertical bars represent standard deviations of the means.

Next, we compared SFC counts between individuals who remained sputum culture positive at completion of 8 weeks of treatment and individuals who were sputum culture negative at completion of 8 weeks of treatment. Forty-eight (82.8%) of 58 individuals were culture negative at 8 weeks, and 10 (17.2%) of 58 remained culture positive. As shown in Figure 2, individual participant RD1 SFC response profiles demonstrated large intersubject and intrasubject variability. Mean SFC counts are shown in Figure 3. Compared with subjects having negative culture results, culture positive subjects had higher mean SFC counts at all time points and for both ESAT-6 and CFP-10. Mean ESAT-6 SFC count was on average 2.23 times higher for the group that was culture positive at completion of 8 weeks of treatment than for the group that was culture negative (IRR 95% CI 1.01 to 4.93, p = 0.048). The mean CFP-10 SFC count was on average 1.51 times higher for the culture positive group compared to the culture negative group, but this difference did not reach statistical significance (IRR 95% CI 0.80 to 2.85, p = 0.20). The mean RD1 SFC count was on average 1.83 times higher for the culture positive group compared to the culture negative group (IRR 95% CI 1.01 to 3.33, p = 0.047); the rate of decline did not differ between the two groups (p = 0.54). At completion of 24 weeks of therapy, the culture positive group had ESAT-6 and CFP-10 counts that were 2.27 (IRR 95% CI 0.82 to 6.31) and 2.36 (IRR 95% CI 0.92 to 6.04) times higher than the culture negative group, but neither of these differences reached statistical significance.

Using qualitative results (Table 2), compared with culture negative subjects, slightly higher percentages of culture positive subjects had ESAT-6 or CFP-10 results classified as reactive at weeks 0, 16, and 24, but differences were not statistically significant.

## Discussion

There has been considerable debate about the immune response to *M. tuberculosis *and correlates of bacterial containment in the human host. It has been postulated that active TB infection is immunosuppressive and that treatment of TB would lead to improved immune function and an increase in antigen-specific IFN-γ production by T cells.[17] Our data support an alternate hypothesis that treatment leads to decreased antigen burden and, in turn, diminished frequency of circulating antigen-specific T cells. In this regard, our findings are consistent with those of several prior studies using precommercial RD1-based interferon gamma release ELISPOT assays. [1,4,18,19] Specifically, Pathan et al. used an RD1-specific ELISPOT assay to evaluate kinetics of T cell responses in 12 active TB patients undergoing treatment.[1] They observed an average 38% decrease in SFCs over a mean follow-up period of 18.6 weeks. Aiken et al. showed that 55% of treated pulmonary TB patients had negative ESAT-6/CFP-10 ELISPOT tests one year after treatment initiation.[19] Some studies using precommercial assays, however, have demonstrated persistently high or positive RD1-specific ELISPOT test results even after treatment; multiple technical or environmental factors may affect assay performance characteristics.[20,21] One study using the commercially-available T-SPOT.*TB *comparing patients early in their TB treatment to patients late in their TB treatment showed significantly higher positive T-SPOT.*TB *proportions in the early-treatment group (83% vs. 17%).[22]

Our findings add strength to the hypothesis that SFC counts directly reflect bacillary burden and, importantly, used the commercially available T-SPOT.*TB *assay. Mean ESAT-6 and summed RD1 SFC counts were statistically significantly higher in the group with positive sputum cultures at week 8 compared with the group having negative sputum cultures at week 8. These differences were apparent at baseline (time 0), and overall rates of decline in RD1 SFC counts did not differ between groups. These data suggest that higher SFC counts in those with positive sputum culture results at week 8 resulted from higher pre-treatment bacillary burdens of *M. tuberculosis *rather than from a slower decline in SFC counts during treatment. In addition, there was an association between ESAT-6 SFC count and presence of lung cavitation – a manifestation of TB disease usually characterized by high bacillary burden.

Additional studies of *M. tuberculosis *exposure, progression from latent to active TB, and treatment of latent TB also support the hypothesis that quantitative ELISPOT readout reflects mycobacterial burden. Specifically, Hill et al. found that, in individuals with positive ELISPOT responses to ESAT-6 and CFP-10, quantitative ELISPOT responses to PPD reflected the infectious load of *M. tuberculosis *as a result of recent exposure.[23] Richeldi et al. reported a newborn child exposed to TB – ELISPOT results turned positive at age 6 months, and were approximately 10-fold higher at age 24 months when the child developed overt TB disease.[24] In another case report, a rising ELISPOT count heralded progression to active TB in a patient with known latent TB.[25] With respect to treatment of latent TB, Ewer et al. used an RD1-specific ELISPOT assay to evaluate kinetics of T cell responses in individuals presumably recently infected with *M. tuberculosis *in the context of a point-source school-associated TB outbreak.[26] Tuberculin skin test-positive students treated with a three-month course of isoniazid and rifampin had an average 68% decline per year in frequencies of RD1 specific SFCs, but no change in frequencies of these cells was observed in untreated individuals. Chee et al. recently demonstrated that among individuals undergoing treatment for latent TB infection, treatment had a significant effect on response to CFP-10 but not on response to ESAT-6 as measured by the T-SPOT.*TB *assay.[27]

While our results and those of others indicate that immunologic responsiveness as measured by RD1-based interferon gamma release ELISPOT assays is dynamic during the course of *M. tuberculosis *disease and its treatment, our results indicate that the test's utility in evaluating or predicting treatment response in individual patients appears poor. Our results do not support this test's use as an early surrogate marker for treatment response in clinical trials. While overall SFC counts declined over the course of treatment, individual patient response profiles (mapping SFC counts over time) were highly variable in our study. Furthermore, the proportion of patients with positive qualitative test results was not different between groups and did not decline over time during treatment. Further studies evaluating this test in subgroups, such as HIV-seropositive patients or patients with solely extrapulmonary disease, may help determine the utility of this test in these special populations. The utility of an RD1-based interferon gamma release ELISPOT assay in combination with other immunologic tests to evaluate treatment response also remains to be studied.

Our study has important limitations. First, we used frozen cells for cost reasons. Freeze-thaw processes may have diminished T cell reactivity, and this could account for the lower-than-expected baseline sensitivity of the T-SPOT.*TB *assay. We cannot exclude the possibility that frozen samples that were stored longest before testing (i.e., baseline samples) could have been most affected by storage and might have given spuriously low results; this could have diminished our ability to detect a decrease in assay responses with treatment. The effects of prolonged freezing could be studied by dividing samples into two aliquots, with one aliquot tested immediately and the other frozen for later testing. Second, we compared T-SPOT.*TB *to a surrogate marker–sputum culture status at completion of eight weeks of treatment–and not to the accepted gold standard of durable TB cure without relapse after treatment. This was done for efficiency and convenience in our study but highlights the need and provides rationale for additional studies of T-SPOT.*TB *responses during long-term follow-up after completion of TB treatment. In our study, only 10% of subjects with a reactive T-SPOT.*TB *test at baseline had a nonreactive test at completion of 24 weeks of treatment. Aiken et al. showed that 55% of treated pulmonary TB patients had negative ESAT-6/CFP-10 ELISPOT tests one year after treatment initiation.[18] It remains to be seen whether, after successful TB treatment, SFC counts return to zero in a substantial majority of patients at time points beyond one year. If this were the case, the test might have utility in predicting or diagnosing relapsed or recurrent TB disease. In our study, there were no subjects with recognized TB treatment failure, and therefore we were not able to evaluate T-SPOT.*TB *responses in this important clinical scenario. We were also unable to assess the kinetics of T cell responses early in treatment, as funding limitations precluded the performance of an 8-week TSPOT-*TB *test. Of note, current manufacturer instructions in the U.S. FDA-approved T-SPOT.*TB *assay classify those with spot counts of 5, 6, or 7 as borderline and recommend retesting. Retesting was not done for three individuals in our study who had spot counts of 5 at baseline, as manufacturer instructions at the time of our study classified them as negative. Using the current classification system, two of the three had positive results upon completion of treatment while one had a negative result, resulting in no substantive change in our overall results or conclusions.

On the other hand, our study has several strengths. First, the lab study was undertaken in a setting in which TB treatment (under direct observation), sputum monitoring, and subject follow-up were standardized and rigorous. In addition, the T-SPOT.*TB *laboratory technologist was not aware of patient status, thereby reducing potential bias. Finally, for each study subject, all T-SPOT.*TB *specimens were thawed and tested at the same time, thereby reducing the impact of potential unrecognized time-related differences in study procedures.

## Conclusion

In conclusion, the correlation between ESAT-6 SFC counts and cavitation on baseline chest radiograph, the decline in SFC counts during treatment, and the observed differences in SFC counts between the group having positive 8-week cultures versus the group having negative 8-week cultures indicate that IFN-γ-producing RD1-specific T cells, as measured by the T-SPOT.*TB *assay, may be directly related to bacterial load in individuals undergoing treatment for pulmonary TB. However, high inter-patient variability in quantitative results as well as lack of reversion to negative of qualitative results in patients at the time of treatment completion, may limit the utility of this test as an early surrogate marker for treatment response. Longitudinal studies with long-term patient follow-up are warranted in order to determine the utility of the T-SPOT.*TB *test for detecting relapse after TB treatment.

## Competing interests

The authors declare that they have no competing interests.

## Authors' contributions

SED conceived of and designed the study and helped to draft the manuscript. KD performed the statistical analyses and drafted the manuscript. NB, MBC, and REC assisted with study design, interpretation of results, and manuscript preparation. SR performed the TSPOT.*TB *laboratory assays. JH, AE, and CL coordinated the study and recruited study participants. All authors read and approved the final manuscript.

## Pre-publication history

The pre-publication history for this paper can be accessed here:

http://www.biomedcentral.com/1471-2334/9/23/prepub
